# Intracisternal vs intraventricular injection of AAV1 result in comparable, widespread transduction of the dog brain

**DOI:** 10.1038/s41434-024-00510-9

**Published:** 2024-12-09

**Authors:** Jacqueline E. Hunter, Charles H. Vite, Caitlyn M. Molony, Patricia A. O’Donnell, John H. Wolfe

**Affiliations:** 1 Research Institute of Children’s Hospital of Philadelphia, Philadelphia, PA USA; 2 W.F. Goodman Center for Comparative Medical Genetics, School of Veterinary Medicine, University of Pennsylvania, Philadelphia, PA 19104 USA; 3 Department of Pediatrics, Perelman School of Medicine, University of Pennsylvania, Philadelphia, PA 19104 USA; 4 Present Address: Department Small Animal Clinical Sciences, College of Veterinary Medicine, University of Florida, Gainesville, FL 32608 USA

**Keywords:** Genetic vectors, Cellular neuroscience

## Abstract

Widespread distribution of transduced brain cells following delivery of AAV vectors into the cerebrospinal fluid (CSF) of the cisterna magna (CM) has been demonstrated in large animal brains. In humans, intraventricular injection is preferred to intracisternal injection for CSF delivery due to the risk of brain stem injury. One study in the dog reported adverse reactions to AAV vectors expressing GFP injected into the lateral ventricle but not when injected into the CM. In contrast, AAV expressing mammalian genes in diseased animals have not triggered adverse responses since many genetic diseases also have compromised immune systems. Differences in circulation of CSF from each site could potentially affect vector spread within the brain, but a direct comparison has not been made using both a mammalian gene and immunologically normal animals. In this study we evaluated the dopamine-2-receptor (D2R) variant D2R80A, which is inactivated for intracellular signaling and has been used as a reporter gene in large animal brains. No adverse reactions to the D2R80A gene were observed from either injection route in normal dogs and both routes resulted in comparable distribution of D2R80A within the brain.

## Introduction

CSF-directed routes of administration are a promising option for delivery of gene therapy to the brain [1]. Previous studies using adeno-associated viral vectors (AAVs) have demonstrated widespread transduction of the large mammalian brain following cisterna magna (CM) injection [2–8], however this delivery method is not routinely used clinically. Intraventricular injection is preferred to intracisternal injection for CSF delivery in humans due to the risk of brain stem injury. Successful CSF gene delivery has been achieved via ICV injection in a variety of species [9–13]. It is unclear, however, whether there is a difference in transduction pattern resulting from the two injection routes. To date, few studies have compared both routes side by side [11, 13–15], and low numbers of test subjects were used in the large animal studies.

We have previously performed many CM injections in cats using GFP vectors to assess vector distribution in the brain without adverse effects [2, 4]. One study in dogs found that GFP vector delivered to the lateral ventricles (LVs) resulted in an encephalitis associated with a T-cell response to GFP [11]. In contrast, other groups have not observed adverse reactions with ICV delivery of GFP vector in dogs [16, 17]. Furthermore, ICV injections using AAVs expressing normal mammalian genes have been used in several studies without incident [11, 17–19]. To avoid the confound of potential toxicity due to the expression of GFP, we compared the distribution of an AAV vector expressing a mammalian gene in normal dog brains by CM or ICV administration. We used an AAV1 encoding a variant of the highly conserved dopamine-2-receptor (D2R), D2R80A which is inactivated for intracellular signaling and has been used as a reporter gene in mice and cats to assess vector distribution without evidence of toxicity [20]. Dogs were chosen because the dog brain has normal anatomical variations including larger lateral ventricular spaces in some animals, that can be determined pre-operatively by MRI to insure that the ICV injection is only into the fluid space. Dog and cat brains have essentially the same structure [21].

## Materials and methods

### Plasmids and AAV production

The gene transfer cis-plasmid containing a rat dopamine-2-receptor (D2R) mutant (D2R80A) gene was expressed from the minimal human GUSB promoter in the pZac vector that included an SV40 splice donor/acceptor signal and the bovine growth hormone polyadenylation signal, as described [20, 22–25]. Recombinant AAV1 vectors were packaged by the University of Pennsylvania Vector Core by triple transfection of HEK293 cells with the AAV cis-plasmid, AAV trans-plasmid containing AAV rep and cap genes, and adenovirus helper plasmid. Vectors were purified using iodixanol gradient ultracentrifugation, and the titers were determined by real time PCR [26].

### Animals and vector injections

All animal care and procedures were in accordance with the Institutional Animal Care and Use Committee at the University of Pennsylvania. MRI was used to assess the size of the lateral ventricles, and animals were sorted into treatment groups based on ventricular size. No blinding was performed during sample collection or processing.

For cisterna magna (CM) injections, dogs at 15–19 weeks of age were anesthetized with i.v. propofol (up to 6 mg/kg). A catheter was placed in the cephalic vein and enough propofol was given to allow intubation. Animals were positioned in right-side lateral recumbency and were anesthetized during the procedure. Using sterile techniques, approximately 0.25 mL of CSF was collected using a 22-gauge needle from the cerebellomedullary cistern. After collecting CSF, AAV1-D2R80A (1.0 × 10^13^ vg/animal, 0.25 mL total volume) was injected over the course of 1 min. A post-injection dwell time of approximately 5 s was used before withdrawing the needle, which was selected based on previous studies utilizing CM injections [2, 4].

For intraventricular (ICV) injections, dogs at 15–19 weeks of age were anesthetized and intubated as described for CM injections. Animals were positioned in a stereotaxic head holder. A 3-cm-long skin incision was made in the dorsal midline of the skin over the skull. Subcutaneous tissue was moved aside. A 25-gauge drill bit was used to drill a hole in the side of the skull corresponding to the larger of the two lateral ventricles. A needle was advanced to the predetermined depth and placement was confirmed by CSF return. Approximately 0.25 mL of CSF was collected, then AAV1-D2R80A (1.0 × 10^13^ vg/animal, 0.25 mL total volume) was injected over the course of 1 minute. A post-injection dwell time of approximately 5 s was also used before withdrawing the needle to avoid reflux up the needle track, based on previous studies utilizing ICV injections [2, 4].

For both procedures, heart and respiratory rates were monitored until full recovery. All animals were injected by the same staff and using the same procedure with the same vector at the same dose. Animals were assessed daily by veterinarians or trained veterinary staff and were euthanized 6 weeks post injection.

### Tissue collection

Euthanasia was performed using an overdose of intravenous barbiturates and transcardially perfused with 0.9% cold saline. Tissues were drop-fixed in 4% paraformaldehyde for 48 h. Euthanasia allowed these brains to be perfused with PBS and immediately transferred to paraformaldehyde to preserve brain morphology.

For cryosectioning, brains were cryoprotected in 30% sucrose, embedded in optimum cutting temperature solution (Sakura, Torrance, CA) and cryosectioned at 40 µm (Leica Microsystems, Wetzlar, Germany).

For serum collection, whole blood was allowed to clot for 30 min at room temperature then centrifuged at 1000 × *g* for 15 min. The supernatant was then aspirated and stored at −80 °C.

### Real-time PCR

Quantitative real time PCR was used to determine the viral genome copies present in the brain. Genomic DNA was extracted from 2–3 sections of each transverse brain block and the vector genome copies were quantified separately at each transverse level. Copies of D2R80A vector genome were quantified using PowerUp SYBR Green Master Mix (Thermo Fisher, Austin, TX). Triplicate samples derived from each DNA pool were used for quantification. The D2R80A primer sequences were as follows: forward: 5’-[ACC TCC CGC GCT TTT CTT AG]-3’, reverse: 5’-[GCT CCA GTT CTG CCT CTC CA]-3’.

### Statistical analysis

Unpaired two-tailed Student’s *t* test was used to determine mean differences between groups. Data are reported as mean ± SEM unless otherwise stated.

## Results and discussion

### D2R80A is well tolerated following either intraventricular or intracisternal injection

One study in normal dogs reported an adverse reaction following ICV injection of a GFP-expressing vector [11]. To test a mammalian gene they injected a therapeutic vector into a LSD diseased dog, however, in only a single animal. Abnormalities in immune response is a feature of many LSDs, thus the lack of an adverse event in the diseased dog could have been due to the underlying pathology. In contrast, other studies have performed ICV injections using GFP-expressing AAVs in both normal and Krabbe disease dogs without incident [16, 17]. The current study was designed to bypass both issues by: 1) using normal dogs with intact immune systems; and 2) to avoid immune responses to the vector transgene by using a mammalian gene encoding a variant of the highly conserved dopamine receptor 2 (D2R), the non-signaling D2R80A, which has been used in PET studies in the cat without adverse effects [20].

Dogs were injected with 1.0 × 10^13^ vg AAV1-D2R80A in pairs (one ICV and one CM) at 15–19 weeks of age, with three pairs injected in total (Table 1). All animals recovered well from surgery and no adverse reactions to the D2R80A gene were observed over the course of the study from either the ICV or CM injections (Table 2). No gross lesions were observed in brains when collected at necropsy. To assess whether expression of D2R80A had any effect on peripheral tissues, serum was collected pre-euthanasia from both injected and noninjected dogs. There were no significant differences between injected and non-injected animals, and both groups had values within the normal range for liver enzymes, bilirubin, and creatinine. These data demonstrate no adverse effects on liver or kidney function following CM or ICV injection of D2R80A (Table 3).

**Table 1:** Experimental design.

Animal	Route	Vector dose (vg/animal)	Vector volume (mL)
1	CM	1.0 × 10^13^	0.25
2	CM	1.0 × 10^13^	0.25
3	CM	1.0 × 10^13^	0.25
4	ICV	1.0 × 10^13^	0.25
5	ICV	1.0 × 10^13^	0.25
6	ICV	1.0 × 10^13^	0.25

**Table 2:** Clinical response to D2R80A injection.

	Age injected (weeks)	Age euthanized (weeks)	Clinical response	Adverse events
Pair 1	15	21	Normal recovery from anesthesia	None
Pair 2	18	24	Normal recovery from anesthesia	None
Pair 3	19	25	Normal recovery from anesthesia	None

**Table 3:** Serum chemistry of dogs used in study.

Test	Reference range	Uninjected		Cisterna magna			Intraventricular			Units
Liver function										
ALT	16–91	38	34	26	61	32	28	39	32	U/L
AST	23–65	42	41	39	41	39	34	38	42	U/L
Alk. Phos.	20–155	148	167	126	91	80	113	96	138	U/L
Kidney function										
BUN	5–30	13	15	17	18	15	16	18	13	mg/dL
Creatinine	0.7–1.8	0.6	0.7	1	0.9	0.8	0.8	0.8	0.6	mg/dL

Blood was collected from both treated and untreated dogs during the study and analyzed for several markers of liver and kidney function. Columns contain values for each animal in a treatment group.

### Comparable widespread transduction after either CM or ICV injection

**Fig. 1:**
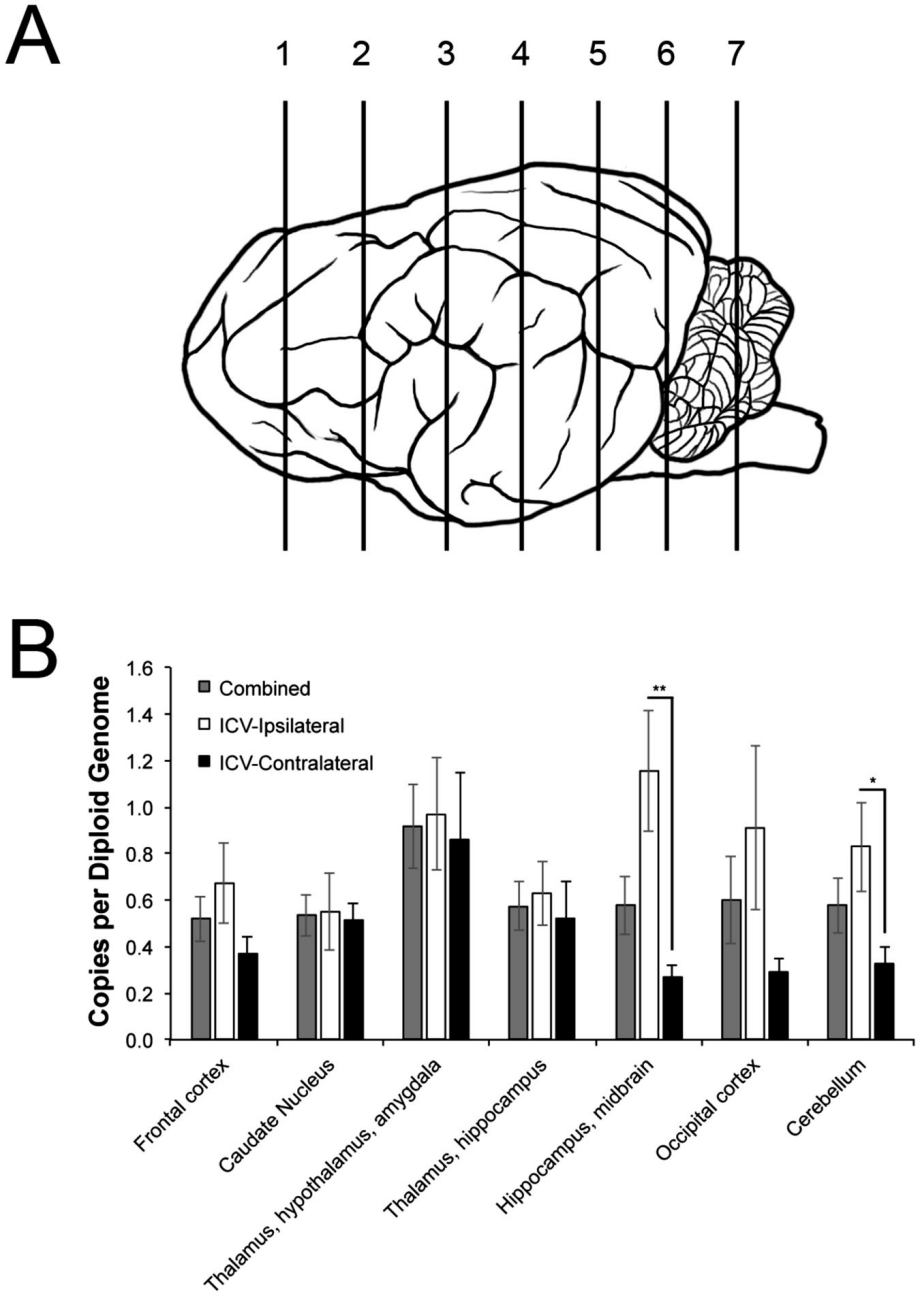
Vector copies per diploid genome in ipsilateral vs contralateral hemispheres in ICV-injected dogs. **A** Approximate location of hemisections analyzed in study. Normal dogs were treated with a single injection of AAV1 expressing D2R80A at a dose of 1.0 × 10^13^ vg either intracisternally (*n* = 3), or intracerebroventricularly (*n* = 3). Numbers correspond to the approximate location of hemisections analyzed and contain the following structures: (1) frontal cortex; (2) caudate nucleus; (3) thalamus, hypothalamus, and amygdala; (4) thalamus and hippocampus; (5) hippocampus and midbrain; (6) occipital cortex; or (7) cerebellum. **B** Copies per diploid genome were measured at several sites along the rostral-to-caudal axis in both ipsilateral and contralateral hemispheres. A similar number of vector genome copies were measured in hemisections collected near the injection site, however greater divergence was seen further from the ventricle, with lower levels in the contralateral side. The average of both hemispheres was used in comparisons to animals injected intracisternally. Error bars represent mean ± SEM. *p < 0.05; **p < 0.01.

Animals were euthanized at 6 weeks post injection, and vector distribution was assessed by qPCR. Several hemisections along the rostral to caudal axis were assayed, the approximate locations are shown in Fig. 1A. Vector transduction after CM injection was symmetrically distributed bilaterally [1, 6]. For the ICV group, hemisections from the injected and contralateral hemispheres were analyzed and the amounts of vector genome per diploid cell genome were averaged for each region to obtain the total transduction for comparison to the CM group. Most structures had similar amounts in the ipsilateral and contralateral hemispheres. Greater divergence was seen in the more rostral and caudal regions, further from the ventricle, with lower levels in the contralateral side (Fig. 1B). Vector distribution was similar between the two treatment groups for all regions tested. Some variability was present in each group, however the difference between the mean copies per diploid genome was not significant for any of the structures (Table 4). The results indicate that similar vector distribution is produced by either injection route, a finding also reported by others [11, 13]. Greater variability was seen in the ICV-injected group, with a greater number of vector genomes detected in the injected hemisphere. This suggests that more consistent distribution may be achieved with CM injection. Although vector copies per diploid genome seem relatively low, the dose used was sufficient to generate widespread GFP expression in normal cat brains [4].

**Table 4:** Mean vector copies per diploid genome for each treatment group.

Brain region(s)	Injection site				*p* value	Significance
	Cisterna magna		Lateral ventricle			
	Copies per diploid genome	SEM	Copies per diploid genome	SEM		
Frontal cortex	0.359	0.016	0.521	0.166	0.386	ns
Caudate nucleus	0.509	0.351	0.532	0.185	0.956	ns
Thalamus, hypothalamus, amygdala	0.441	0.193	0.916	0.300	0.254	ns
Thalamus, hippocampus	0.454	0.201	0.575	0.294	0.751	ns
Hippocampus, midbrain	0.582	0.368	0.589	0.231	0.989	ns
Occipital cortex	0.436	0.170	0.602	0.339	0.686	ns
Cerebellum	0.292	0.188	0.577	0.267	0.433	ns

AAV1-D2R80A vector genome copies in normal dogs (*n* = 3 for each group) were measured in samples taken from several points along the rostral-to-caudal axis of the brain. For the ICV-injected group, the averages of the contra- and ipsi-lateral hemispheres were used for comparison to the CM-injected group. ns = not significant.

The results of this study indicate that comparable total transduction can be achieved with either CM or ICV injection. Although ICV may be preferred by surgeons over CM for CSF delivery in human patients, CM delivery via a lumbar catheter has been shown to be feasible and safe in large animals [3, 6, 15] and has been used in a clinical trial of the LSD Tay-Sachs disease without adverse effect [27]. While few studies directly compare these delivery methods in large animal brains, our findings are consistent with what others have observed [11, 13, 15, 16], with the current study performed using a conserved mammalian gene in normal animals with an intact immune system.

## Data Availability

Data generated and analyzed during this study can be found within the published article. Raw data can be provided by the corresponding author upon reasonable request.
